# Effects of insulin resistance on left ventricular hypertrophy in patients with CKD stage 1–3

**DOI:** 10.1007/s11255-014-0720-3

**Published:** 2014-05-18

**Authors:** Cheng-jun Wang, Xiao-rong Bao, Guo-wei Du, Yu Wang, Kai Chen, Ma-li Shen, Li-zhen Wang

**Affiliations:** Department of Nephrology, Jinshan Hospital, Fudan University, Long Route 1508, Jinshan, Shanghai, 201508 China

**Keywords:** Chronic kidney disease, Insulin resistance, Left ventricular hypertrophy

## Abstract

**Background:**

Left ventricular hypertrophy (LVH) existed in patients with early stage chronic kidney disease (CKD). But whether insulin resistance (IR) exists in these patients and has some definite relationship with LVH, is unknown.

**Methods:**

Homeostatic model method was used for detecting homeostasis model assessment of insulin resistance (HOMA-IR) in 336 subjects including 286 patients with early stage CKD and 50 control subjects, and HOMA-IR and other clinical data in all subjects were obtained based on standard methods. Then, the relationship between LVH, IR and other relevant clinical data were analyzed.

**Results:**

IR and LVH existed in early stage CKD patients. The prevalence of LVH in patients with IR was significantly higher than those without, and patients with LVH had a higher prevalence of IR than those without. The patients with IR or LVH had lower levels of e-GFR, hemoglobin (Hb) and total cholesterol, while higher levels of blood urea nitrogen (BUN), serum creatinine (Scr), intact parathyroid hormone (iPTH), CRP and systolic blood pressure (SBP). HOMA-IR had positive correlations with left ventricular mass index (LVMI). HOMA-IR and LVMI had positive correlations with BUN, Scr, iPTH and CRP, but negative with e-GFR and Hb. Multiple linear stepwise regression analysis showed that e-GFR, FINS, Hb and SBP enter the regression equation. Binary unconditional logistic regression analysis indicated that the main risk factors for LVH were CKD and IR (*P* < 0.05, respectively).

**Conclusion:**

Both IR and LVH existed in early stage CKD patients and were more severe with the development of CKD. IR had a significant correlation with LVH. Furthermore, decline of e-GFR, hypertension and anemia were also associated with both IR and LVH and may have some effects in the mechanism of IR on the development of LVH.

## Introduction

Cardiovascular disease (CVD) is the most common complication in patients with chronic kidney disease (CKD) [1–5] and also is the main cause of mortality in these patients. Recent studies have shown that CVD, including left ventricular hypertrophy (LVH), existed in early stage CKD patients, even when their estimated glomerular filtration rate (eGFR) is within the normal range. But the factors for the development of LVH remain unclear. Insulin resistance (IR), commonly companied by compensatory hyperinsulinemia, has been demonstrated to exist in patients with end-stage renal disease and play an important role in the development of uremic complications, such as hypertension, dyslipidemia, atherosclerosis and CVD, including LVH, heart failure and all-cause mortality in this population [3–9]. But whether IR exists in patients with early stage of CKD, and whether IR has some definite relationship with LVH, is unknown. The aim of this study was to investigate whether IR exists and its relationship with LVH in early stage CKD patients, in order to take some steps to reduce the prevalence and severity of LVH and to improve the prognosis.

## Materials and methods

### Patients and controls

Two-hundred and eighty-six patients with CKD stage 1–3, 145 males, 141 females, age ranged from 14 to 88 years old, initial hospitalized, never accepting drugs affecting blood pressure or plasma glucose before, in the department of nephrology, Jinshan Hospital, Fudan University during the period of January 2010 to December 2012 (CKD group) were enrolled. All patients were diagnosed according to the criteria from kidney disease outcomes quality initiative (K/DOQI) and had a stable renal function at least 3 months recently, and their eGFR were above 30 ml/min 1.73/m^2^. The causes of CKD included chronic glomerulonephritis (CGN, 186), IgA nephropathy (IgAN, 86) and chronic tubulointerstitial nephropathy (CTIN, 3); and autosomal dominant polycystic kidney disease (ADPKD, 11). All patients with CGN, IgAN or CTIN were diagnosed by renal biopsy.

The exclusion criteria included history of diabetes mellitus, hypertension or other CVD; treatment with glucocorticoids in the past 6 months, or with agents, such as angiotensin converting enzyme antagonists (ACEIs), angiotensin receptor blockers (ARBs), calcium channel blockers (CCBs) or diuretics in the past 3 months; infection, surgery, trauma history in the past 1 month; rapid decline of renal function in the past 3 month; malignancy or pregnancy.

The patients were divided into three groups according to the eGFR, namely Group A (*n* = 141): eGFR ≥90 ml/min 1.73/m^2^; Group B (*n* = 72): eGFR = (60–89) ml/min 1.73/m^2^; Group C (*n* = 73): eGFR = (30–59) ml/min 1.73/m^2^.

Fifty healthy subjects, 24 males, 26 females were enrolled in this study to serve as control group. They came from the examination center of our hospital in the same period, were no overweight, obesity or thinness, had no history of medical disease (such as diabetes mellitus, hypertension, other CVD, and kidney disease), and were of normal renal function, and urinary albumin creatinine ratio (ACR) <3 mg/mmol (spot urine), and never receiving medical treatment.

All studies were approved by the Ethics Committee of Jinshan Hospital, Fudan University, and all patients gave written informed consent.

### Clinical data and laboratory test

Data on demographic characteristics, medical history, current medications and blood samples were collected from all subjects at the time of enrollment. Blood was collected from subjects after overnight fasting for at least 10 h. Blood urea nitrogen (BUN), serum creatinine (Scr), serum uric acid (UA), hemoglobin (Hb), fasting plasma glucose (FPG), 2-h postprandial plasma glucose (2hPG), triglycerides (TG), total cholesterol (Tch), high-density lipoprotein cholesterol (HDL-c), low-density lipoprotein cholesterol (LDL-c), calcium (Ca), phosphate (P) and urinary creatinine (Ucr) were measured with P800 automatic biochemical analyzer (the Roche, Beijing, China) and the supporting reagent. Fasting insulin (FINS) and 2-h postprandial insulin (2hINS) were measured with i2000 chemiluminescence analyzer (Abbott, USA). Twenty-four-hour urinary protein (UP) and spot urine microalbuminuria (UMA) were measured with BN ProSpec special protein analyzer (Behring, Shanghai, China). Intact parathyroid hormone (iPTH) concentrations were measured with the Roche Elecsys parathyroid hormone assay (Roche, Indianapolis, IN, USA). C-reactive protein concentration was measured with enzyme-linked immunosorbent assays (CRP, R&D, USA). All other blood tests were done in Clinical Examination Laboratory of Jinshan Hospital of Fudan University. Laboratory quality control was performed daily and met the quality control standards of clinical laboratory center of Shanghai. All data were measured based on standard clinical examination methods.

### Blood pressure measurement

All participants avoided tobacco and caffeine for at least 30 min, then emptied their bladder, quietly rested on chair for at least 5 min in a quiet environment. Before blood measurement, radial artery palpation were performed to estimate systolic pressure; then, the cuffs were inflated to a value 20 mmHg higher than the level that obliterates the radial pulse and deflated at a rate of 3–5 mmHg/s. Blood pressures were measured twice in both arms by a mercury sphygmomanometer after 5 min of standing at two visits with the participants seated, the back supported, and the arm bare and at heart level with an appropriate cuff. According to Korotkoff method, the first loud flop represented systolic blood pressure (SBP), the sound disappearance or suddenly became boring represented diastolic blood pressure, then took the average of two readings as the measurement results.

### Echocardiography

All subjects underwent echocardiographic measurements in left lateral and supine position using Color Doppler ultrasound device type Vivid-7 (company GE, USA) with probe frequency of 2.5 MHz. The M-mode image obtained and the data, such as left ventricular internal diameter (LVDd), interventricular septal thickness (IVST) and left ventricular posterior wall thickness (PWT) in end-diastolic were measured under the guidance of the axis of the left ventricular segments of the two-dimensional image in the parasternal long axis for three consecutive cardiac cycles, averaged. The work was interpreted by a single reviewer who was blinded to this study.

### Definition of variables

1. Body weight index (body mass index, BMI) = body weight (kg)/height^2^ (m^2^). Overweight and obese was defined according to the Working Group on Obesity in China (WGOC, 6):
  Overweight was defined as BMI ≥24 kg/m^2^ and obese BMI ≥28 kg/m^2^.
2. Body surface area (BSA) was estimated by Ye’ equations [10]:
  BSA (m^2^) = 0.607 × height (m) + 0.0127 × weight (kg) − 0.0698 (male).
  BSA (m^2^) = 0.568 × height (m) + 0.0126 × weight (kg) − 0.0461 (female).
3. eGFR was estimated by MDRD equations [1].
  eGFR (ml/min/1.73 m^2^) = 186 (Scr in µmol/l × 0.011312)^−1.154^ × (age)^−0.203^ × (0.742 if female).
4. Homeostasis model assessment of insulin resistance (HOMA-IR) was estimated by Matthews’ equations [11]:
  HOMA-IR = fasting insulin (μIU/ml) × fasting glucose (mmol/l)/22.5.
  The HOMA-IR value of all CKD participants was 2.16 ± 0.53, while that of the healthy ones was 1.87 ± 0.21.
  IR was defined as follows: log HOMA-IR ≥2.287, the cutoff point being a log HOMA-IR value of $\bar{x}$ + 2*s* (the 95th ‰) in all control subjects [12].
5. ACR = UMA (mg)/Ucr (mmol), spot measured.
6. Hypertension was defined as SBP >140 mmHg or/and DBP >90 mmHg.
7. Left ventricular mass index (LVMI) was calculated with the Devereux formulation [13, 14]:
  Left ventricular mass (LVM) = 0.8 × 1.04 × [(LVDd + IVST + PWT)^3^ − LVDd^3^] + 0.6 (g).
  LVMI = LVM/BSA.
  LVH was defined as LVMI >125 g/m^2^ for men and >110 g/m^2^ for women [15, 16].

### Statistical analysis

All of the statistical analyses were performed with Statistical Package for Social Sciences, version 11.5 (SPSS Inc., Chicago, IL, USA). In brief, continuous data with normal distribution and homogeneity of variance determined by Shapiro–Wilk test were expressed as mean ± SD ($\bar{x}$ ± *s*), those with abnormal distribution or variance homogeneity were logarithmically transformed before analysis and treated as normally distributed if met the above conditions. Two-group normally distributed data were compared using independent sample *t* test, and multi-group data were compared using single factor analysis of variance (one way ANOVA). Count data were expressed as the number of cases (ratio or percentage) and compared using chi-squared test (χ^2^ test). All multiple testing was corrected using Bonferroni correction. Single factor linear correlation analysis and multiple linear stepwise regression analysis were used to explore the associations between examined continuous variables with parametric distribution if the plot showed a linear relationship. Binary unconditional logistic regression analysis was used to explore the main risk factors. *P* values <0.05 were considered as statistical significance.

## Results

### Demographic and clinical characteristics of participants

There were no differences of gender ratio, the levels of age and BMI in the subjects between the groups (*P* > 0.05, respectively, Table 1).

**Table 1:** Description of participants by level of kidney function

Variables	Control group	Group A	Group B	Group C	*F* or *χ* ^2^	*P* for trend
*N*	50	141	72	73	–	–
Female	26 (52.0 %)	77 (%)	32 (54.6 %)	32 (44.4 %)	3.265	0.353
Age (years)	49.48 ± 10.54	49.87 ± 14.53	51.10 ± 14.87	48.33 ± 17.59	0.430	0.732
BMI (kg/m^2^)	23.49 ± 3.91	23.61 ± 3.97	24.36 ± 3.61	23.28 ± 2.75	1.184	0.361
eGFR (ml/min 1.73/m^2^)	123.16 ± 12.31	121.60 ± 24.82	75.55 ± 8.46^▲^	43.01 ± 8.09^◆^	389.372	**<0.001**
Hb (g/l)	136.56 ± 10.95	130.98 ± 22.92	130.24 ± 18.21	118.65 ± 21.64^★,▲,◆^	9.100	**<0.001**
Ca (mmol/l)	–	2.15 ± 0.19	2.16 ± 0.18	2.13 ± 0.16	0.395	0.647
P (mmol/l)	–	1.28 ± 0.43	1.19 ± 0.21	1.18 ± 0.21	2.238	0.109
Log iPTH (µg/l)	3.25 ± 0.58	3.61 ± 0.41^★^	3.82 ± 0.33^△^	4.10 ± 0.77^◆^	29.644	**<0.001**
CRP (mg/l)	–	2.68 ± 6.40	5.23 ± 9.87	8.12 ± 9.82^▲^	10.454	**<0.001**
ACR (mg/mmol)	–	117.32 ± 58.28	98.30 ± 48.20	641.57 ± 335.52	1.591	0.205
TG (mmol/l)	1.95 ± 0.89	1.86 ± 0.87	1.73 ± 0.65	1.77 ± 0.83	0.374	0.772
Log-Tch	1.55 ± 0.47	1.59 ± 0.35	1.61 ± 0.25	1.64 ± 0.45	0.989	0.414
HDL (mmol/l)	1.25 ± 0.33	1.17 ± 0.49	1.06 ± 0.35	1.04 ± 0.34^★^	3.448	0.017
Log LDL (mmol/l)	1.00 ± 0.49	0.97 ± 0.41	1.11 ± 0.34	1.01 ± 0.42	1.774	0.152
SBP (mmHg)	117.04 ± 7.28	126.35 ± 11.45^★^	131.69 ± 18.69	141.55 ± 19.34^◆^	30.610	**<0.001**
DBP (mmHg)	75.32 ± 6.21	77.59 ± 8.80	81.19 ± 9.93^★^	84.11 ± 12.09^★,▲^	11.496	**<0.001**
Prevalence of hypertension	0 (0.0 %)	14 (9.9 %)	46 (31.9 %)	18 (67.1 %)	103.02	**<0.001**

Bold values indicate statistically significant

Compared with the control group, ^★^ *P* < 0.01

Compared with Group A, ^△^ *P* < 0.05; ^▲^ *P* < 0.01

Compared with Group B, ^◆^ *P* < 0.01

The level of Log iPTH and the concentration of CRP in CKD patients were higher than those in normal control subjects and increased with the development of CKD (*P* < 0.001, respectively, Table 1); contrastively, the concentration of Hb was lower than those in normal control subjects and declined with the development of CKD (*P* < 0.001). There was no difference of the concentration of ACR in the subjects between the groups (*P* > 0.05, Table 1).

Lipid profiles as important component of the MetS were reported in Table 1. It showed an increasing trend of Tch and a decreasing of HDL with the decline of eGFR in CKD patients. The concentrations of HDL was significantly declined in patients with CKD 3 compared with the normal control group (*P* < 0.01), while other data of lipid profiles had no differences between the groups either (Table 1).

The prevalence of hypertension, the levels of SBP and DBP in patients with CKD were higher than those in normal control subjects and increased with the development of CKD (*P* < 0.001, respectively, Table 1).

### IR and LVH in participants

The prevalence of IR, the levels of log HOMA-IR, the concentrations of FPG, FINS, 2hPG and 2hINS were significantly increased in early CKD patients, compared with the control group (*P* < 0.001, respectively, Table 2); both the value of log HOMA-IR and the prevalence of IR were increased with the decline of eGFR in CKD patients.

**Table 2:** Glucose metabolism and echocardiography results of participants

Variables	Control group	Group A	Group B	Group C	*F* or *χ* ^2^	*P* for trend
Prevalence of IR	0 (0.0 %)	31 (22.0 %)	29 (40.3 %)	68 (93.2 %)	140.258	**<0.001**
Log HOMA-IR	1.87 ± 0.21	1.89 ± 0.44^☆^	2.20 ± 0.48^▲^	2.70 ± 0.31^◆^	70.358	**<0.001**
FPG (mmol/l)	4.55 ± 0.28	5.06 ± 0.60^★^	5.65 ± 0.63^▲^	5.96 ± 0.51^◆^	83.971	**<0.001**
FINS (µIU/l)	29.65 ± 3.17	32.64 ± 15.56^☆^	39.80 ± 18.32^△^	59.03 ± 18.83^◆^	44.632	**<0.001**
2hPG (mmol/l)	5.26 ± 0.67	6.47 ± 1.41^★^	7.23 ± 1.54^▲^	8.46 ± 1.30^◆^	65.492	**<0.001**
2hINS (µIU/l)	37.06 ± 6.75	52.80 ± 24.72^★^	50.26 ± 19.26	63.02 ± 23.10^◆^	14.745	**<0.001**
Prevalence of LVH	0 (0 %)	5 (3.5 %)	6 (8.3 %)	41 (56.2 %)	119.693	**<0.001**
LVMI (g/m^2^)	71.11 ± 20.85	79.83 ± 17.99	92.45 ± 15.90^▲^	122.71 ± 31.02^◆^	79.769	**<0.001**
LVDd (mm)	45.92 ± 2.74	47.77 ± 3.23^★^	49.04 ± 2.56^△^	51.86 ± 3.92^◆^	40.467	**<0.001**
IVST (mm)	8.24 ± 1.19	8.52 ± 1.13	9.43 ± 1.10^▲^	10.77 ± 1.76^◆^	58.263	**<0.001**
PWT (mm)	8.081.21	8.24 ± 1.05	9.11 ± 1.12^▲^	9.95 ± 1.36^▲^	42.560	**<0.001**

Bold values indicate statistically significant

Compared with the control group, ^☆^ *P* < 0.05; ^★^ *P* < 0.01

Compared with Group A, ^△^ *P* < 0.05; ^▲^ *P* < 0.01

Compared with Group B, ^◆^ *P* < 0.01

The indicators used to assess LVH, the prevalence of LVH, the levels of LVMI, LVDd, IVST and PWT in patients with CKD were higher than those in normal control subjects and increased with the development of CKD (*P* < 0.001, respectively, Table 2).

### Relationship between IR and LVH

CKD patients were divided into two groups according to the HOMA-IR, namely Group non-IR (*n* = 158): log HOMA-IR <2.287; Group IR (*n* = 128): log HOMA-IR ≥2.287.

The prevalence of LVH, the concentration of BUN, Scr, iPTH and CRP, the level of LVMI, LVDd, IVST, PWT, SBP and DBP, and the prevalence of hypertension in CKD patients with IR were higher than those without IR; contrastively, eGFR and the concentrations of Hb and HDL in CKD patients with IR were lower than those without IR (*P* < 0.05, respectively, Table 3).

**Table 3:** Laboratory data, blood pressure and echocardiogram results of the 286 CKD subjects according to the HOMA-IR

Variables	Group non-IR	Group IR	*t* or *χ* ^2^	*P* for trend
*N*	158	128	–	–
Prevalence of LVH	8 (5.1 %)	44 (34.4 %)	40.841	**<0.001**
LVMI	83.80 ± 19.71	106.42 ± 31.22	−7.748	**<0.001**
LVDd	48.08 ± 3.14	50.44 ± 3.86	−5.704	**<0.001**
IVST	8.79 ± 1.28	9.98 ± 1.73	−6.666	**<0.001**
PWT	8.49 ± 1.20	9.40 ± 1.36	−0.606	**<0.001**
eGFR	107.51 ± 32.90	68.27 ± 32.51	10.082	**<0.001**
BUN	5.56 ± 1.81	7.19 ± 2.91	−5.770	**<0.001**
Scr	70.82 ± 22.30	111.84 ± 44.02	−10.270	**<0.001**
Hb	130.18 ± 22.82	124.51 ± 20.79	2.153	**0.032**
iPTH	43.21 ± 15.66	62.36 ± 30.07	−4.540	**<0.001**
CRP	3.26 ± 1.40	6.51 ± 3.43	−3.230	**0.001**
Tch	5.26 ± 2.23	5.07 ± 1.84	0.750	**0**.**454**
HDL	1.16 ± 0.49	1.05 ± 0.32	2.061	**0.040**
Prevalence of hypertension	32 (20.3 %)	54 (42.2 %)	16.179	**<0.001**
SBP	128.47 ± 15.34	135.42 ± 17.93	−3.533	**<0.001**
DBP	78.94 ± 9.68	81.67 ± 10.97	−2.253	**0.028**

Bold values indicate statistically significant

CKD patients were divided into two groups according to the LVMI, namely Group non-LVH (*n* = 234), and Group LVH (*n* = 52).

The prevalence of IR, HOMA-IR, the concentration of FPG, FINS, 2hPG, 2hINS, BUN, Scr, iPTH and CRP, the level of SBP, the prevalence of hypertension in CKD patients with LVH were higher than those without LVH; contrastively, eGFR, the concentrations of Hb and Tch in CKD patients with LVH were lower than those without LVH (*P* < 0.05, respectively, Table 4).

**Table 4:** Laboratory data, blood pressure and glucose metabolism results of the 286 CKD subjects according to the LVMI

Variables	Group non-LVH	Group LVH	*t* or *χ* ^2^	*P* for trend
*N*	234	52	–	–
IR	84 (35.9 %)	44 (84.6 %)	40.841	**<0.001**
HOMA-IR	2.06 ± 0.49	2.69 ± 0.46	−8.521	**<0.001**
FPG	5.31 ± 0.68	5.95 ± 0.58	−6.281	**<0.001**
FINS	36.97 ± 17.01	60.11 ± 22.88	−8.289	**<0.001**
2hPG	6.95 ± 1.58	8.17 ± 1.49	−5.099	**<0.001**
2hINS	52.69 ± 22.87	64.13 ± 24.19	−3.227	**0.001**
Tch	5.30 ± 2.18	4.58 ± 1.57	2.206	**0.028**
HDL	1.13 ± 0.44	1.04 ± 0.29	1.243	**0**.**215**
eGFR	98.62 ± 34.96	50.90 ± 24.83	9.330	**<0.001**
BUN	5.93 ± 2.20	7.91 ± 3.07	−5.445	**<0.001**
Scr	79.61 ± 31.02	132.23 ± 44.53	−10.140	**<0.001**
Hb	130.61 ± 21.33	114.22 ± 20.46	5.001	**<0.001**
Log iPTH	**3**.**70** ± **0**.**47**	**4**.**17** ± **0**.**71**	−0.599	**<0.001**
CRP	3.91 ± 1.99	8.34 ± 4.11	−3.429	**0.001**
SBP	129.65 ± 16.88	140.27 ± 14.03	−4.222	**<0.001**
DBP	79.60 ± 9.99	82.66 ± 11.62	−1.937	**0**.**054**
Hypertension	58 (24.8 %)	28 (53.8 %)	17.086	**<0.001**

Bold values indicate statistically significant

Pearson’s correlation analysis revealed that HOMA-IR was independently associated with eGFR, BUN, Scr, Hb, iPTH, CRP, LVMI, LVDd, IVST and PWT (*r* = −0.559, 0.352, 0.546, −0.175, 0.329, 0.257, 0.491, 0.354, 0.457, 0.407; *P* < 0.001, respectively).

Pearson’s correlation analysis revealed that LVMI was independently associated with HOMA-IR, FPG, FINS, 2hPG, 2hINS, eGFR, BUN, Scr, Hb, iPTH and CRP (*r* = 0.491, 0.395, 0.456, 0.350, 0.251, −0.603, 0.439, 0.624, −0.294, 0.383, 0.264; *P* < 0.001, respectively).

The analysis also included anthropometric parameters and other biochemical indexes, which were excluded during correlation analysis.

### Risk factors of LVH in CKD patients

Multiple linear stepwise regression analysis revealed that LVMI was independently associated with eGFR, FINS, SBP and Hb in all CKD subjects. The analysis included HOMA-IR, FPG, FINS, 2hPG, 2hINS, eGFR, BUN, Scr, Hb, iPTH and CRP, which were excluded during regression analysis (*P* < 0.05, respectively, the value of pseudo *R* ^2^ was 0.452, Table 5).

**Table 5:** Multiple linear stepwise regression analysis showing variables independently associated with LVMI

Model	Unstandardized coefficients *β*	Standardized coefficients *β*	*t*	*P*	95 % CI
Constant	119.746		8.580	<0.001	92.270–147.223
eGFR	−0.327	−0.448	−8.381	<0.001	−0.4.404 to −0.250
FINS	0.251	0.183	3.508	0.001	0.110–0.391
Hb	−0.237	−0.188	−4.031	<0.001	−0.353 to −0.121
SBP	0.178	0.108	2.244	0.026	0.022–0.334

Dependent variable: LVMI

Binary unconditional logistic regression analysis indicated that the main risk factors were CKD and IR (*P* < 0.05, respectively, main effects model, the value of model chi-square was 112.680, and that of Cox and Snell pseudo *R* ^2^ was 0.285, Table 6). The analysis also included thinness (BMI <24 kg/m^2^), overweight (BMI ≥24 kg/m^2^), obesity (BMI ≥28 kg/m^2^), dyslipidemia (TC ≥6.22 mmol/l, and/or TG ≥2.26 mmol/l, and/or LDL-C ≥4.14 mmol/l, and/or HDL-C <1.04 mmol/l), hyperuricemia (>416 μmol/l, if male, >357 μmo1/l, if female) and hypertension (SBP ≥140 mmHg and/or DBP ≥90 mmHg), which were excluded during logistic regression analysis.

**Table 6:** Binary unconditional logistic regression analysis for the main risk factors of LVH

Independent variables	Standardized coefficients (*β*)	SE	Wald	OR	*P*	*B* (95 % CI)
Constant	−5.787	0.706	67.222		<0.001	
CKD	1.852	0.274	45.794	6.372	<0.001	3.727–10.895
IR	1.664	0.511	10.623	5.280	0.001	1.941–14.361

Dependent variable: LVH

## Discussion

It is well known that kidney is an organ not only excreting for the end products of metabolism, but also playing an important role in metabolism and endocrine. CKD is associated with galaxies of physiological and metabolic disturbances, which are associated to LVH. IR, an abnormal glucose metabolism, clinically defined as the inability of a known quantity of insulin to increase glucose uptake and utilization in an individual as much as it does in a normal population [17], mainly performing a decreased sensitivity to insulin regulation of glucose metabolism and compensatory hyperinsulinemia may be one of its main performance.

Studies have revealed that IR and hyperinsulinemia exist in patients with end-stage renal disease and are closely related to the development of cardiovascular complications, the main and death cause in this population. Recent studies showed that both IR and LVH existed in patients with early stage CKD, even when the GFR was within the normal range [18–22]; however, whether there is a relationship between IR and LVH was rarely reported. This study aims to explore the relationship between IR and LVH, and its mechanism in patients with CKD 1–3 stage provide the theoretical basis for the prevention and treatment of cardiovascular complications in patients with early CKD.

Our data present that IR existed in patients with early stage CKD and got more obvious with the development of kidney injure, which is consistent with previous studies [7, 23, 24]. IR as the central component of the metabolic syndrome often coexisted with hypertension in the early stage CKD patients; however, dyslipidemia, obesity and hyperuricemia is not always the case, compared with the general population. This interesting phenomenon may be derived from reduced muscle activity inpatients with CKD [20, 25–28]. The concentration of FINS increased more significant than other indicators mentioned above in patients with early stage CKD, compared with the general population. It showed that hyperinsulinemia was one of the most important compensatory performances. In view of obese, dyslipidemia and hyperuricemia were not significant in this population; it revealed that significantly reduced muscle activity may mainly correlation to the development of IR.

Our study showed that IR correlated with renal dysfunction, hypertension, anemia, micro-inflammation and hyperparathyroidism. Previous studies have still shown that IR was associated with over-activation of renin–angiotensin–aldosterone system (RAAS) and sympathetic nervous system, and sodium retention. All of these factors above-mentioned can induce in the emergence and development of IR, cause hypertension and endothelial dysfunction, and cause LVH in turn. Previous studies indicated that malnutrition and micro-inflammation, oxidative stress, endoplasmic reticulum stress, metabolic acidosis, vitamin D deficiency and secondary hyperparathyroidism, depressed serum erythropoietin and anemia, and suppressors of cytokine signaling all cause IR by suppressing insulin receptor-PI3K-Akt pathways in CKD [27].

IR can cause hypertension in the early stage CKD patients may through various mechanisms including: (1) IR and HINS, independently of changes in glycemia, can cause sympathetic over-activity with a substantial increase in circulating noradrenaline concentration then increase peripheral vascular resistance [29, 30]. (2) IR leads to increased production of renin and angiotensinogen activates RAAS and other neurohormonal mediators of hypertension, thus participating in blood pressure rises. (3) IR and HINS can increase sodium reabsorption by renal tubular and sodium retention [31, 32] favoring expansion of extracellular fluid volume, which may predispose to hypertension [33]. (4) IR and HINS can activate mediators of inflammation in the visceral fat, liver and muscle then impair the production and release of NO and other vasodilators [34], favor the production of endothelin-1 and the vasoconstrictive and mitogenic responses on the vascular wall [35, 36], diminish endothelium-dependent vasomotion, decrease vasodilator capacity significantly, and promote the migration and proliferation of vascular smooth muscle cells, causing atherosclerosis [37]. Linear regression analysis indicated that e-GFR, FINS, Hb and SBP enter the regression equation, in which LVMI was an independent variable, and logistic regression analysis (LVH was an independent variable) indicated that the main risk factors were CKD and IR. So, we thought that IR was one of the most significant risk factors for LVH, and hyperinsulinemia played a decisive role in the process. Blood pressure increased with CKD progress and was remarkable particularly in CKD3 patients, whose SBP even reaches hypertension diagnostic criteria (they had no previous history of hypertension and related family history and other risk factors. We still thought they suffered from renal hypertension rather than essential, and enrolled them into CKD group). Based on the above causes and mechanisms explore, we believe that hypertension plays a vital role in the cause of IR led to LVH.

Our study revealed that micro-inflammation was evident in the early CKD and was correlation with IR, which is consistent with previous studies [38]. Previous studies have indicated that a complex network of *nutritional* and metabolic alterations underlies CKD, including micro-inflammation, oxidative stress, IR and protein energy wasting, and IR is linked to protein energy wasting and malnutrition [20, 39, 40]. Micro-inflammation developing in patients with CKD may primarily via increased production of proinflammatory cytokines, such as CRP, tumor necrosis factor alpha (TNF-α), interleukin-6 (IL-6) and interleukin-1 beta (IL-1β) [41]. Malnutrition and inflammation would lead to atherosclerosis, namely malnutrition–inflammation–atherosclerosis syndrome. Atherosclerosis, decrease arterial distensibility and arterial compliance lead to increased SBP, resulting in LVH, and a risk factor for CVD. Therefore, metabolic and cardiovascular complications of CKD may be a consequence of abnormal insulin action [42]. Malnutrition–inflammation symptoms often led to reduced quality of life and high mortality in patients with end-stage renal disease. In this specific pathophysiological condition, risk factors for CVD closely correlated with malnutrition involved low cholesterol and so on, rather than “over-nutrition” performance-related high cholesterol, it is known as “reverse epidemiology phenomenon”. Our study revealed that malnutrition and low cholesterol existed in early CKD [43]. It remains further study.

Our study revealed that the concentration of Hb was lower than that in the normal control populations. Anemia may cause sympathetic nerve activity, which is linked to IR and hypertension, increase heart rate and cardiac output, thereby increasing arterial capacity and left ventricular wall tension then cause LVH.

Our study showed that the concentration of iPTH was higher than that in the normal control populations and increased with the declined of eGFR. The mechanisms through which excess PTH blunts insulin sensitivity are still uncertain, but medical treatment of hyperparathyroidism in patients with CKD could lead to correction of glucose intolerance [44].

In summary, both IR and LVH existed in early CKD patients and were more severe with the development of early stage CKD. IR had a significant correlation with LVH, and it may be an important risk factor for the development of LVH. Furthermore, the decline of eGFR, hypertension, anemia and hyperparathyroidism were also associated with both IR and LVH and may have some effects in the mechanism of IR on the development of LVH.

Since this study was a cross-sectional analysis, integrity, accuracy and controllability of data may be affected to some extent. In addition, small sample size led to inadequate power and use of a heterogeneous group of patients with CKD were limitations. It remains to be further prospective study to explore the relationship between IR and LVH in patients with CKD 1–3.
